# Increased circulating CD4^+^FOXP3^+^ T cells associate with early relapse following autologous hematopoietic stem cell transplantation in multiple myeloma patients

**DOI:** 10.18632/oncotarget.25553

**Published:** 2018-06-05

**Authors:** Egor V. Batorov, Marina A. Tikhonova, Natalia V. Pronkina, Irina V. Kryuchkova, Vera V. Sergeevicheva, Svetlana A. Sizikova, Galina Y. Ushakova, Tatiana A. Aristova, Dariya S. Batorova, Elena V. Menyaeva, Andrey V. Gilevich, Ekaterina Y. Shevela, Alexander A. Ostanin, Elena R. Chernykh

**Affiliations:** ^1^ Laboratory of Cellular Immunotherapy, Research Institute of Fundamental and Clinical Immunology, Novosibirsk, Russia; ^2^ Laboratory of Clinical Immunology, Research Institute of Fundamental and Clinical Immunology, Novosibirsk, Russia; ^3^ Department of Hematology and Bone Marrow Transplantation, Research Institute of Fundamental and Clinical Immunology, Novosibirsk, Russia; ^4^ Clinical Diagnostic Laboratory, Research Institute of Fundamental and Clinical Immunology, Novosibirsk, Russia; ^5^ Intensive Care Unit, Research Institute of Fundamental and Clinical Immunology, Novosibirsk, Russia

**Keywords:** CD4^+^FOXP3^+^, autologous hematopoietic stem cell transplantation, immune recovery, multiple myeloma, relapse

## Abstract

We investigated dynamics of CD4^+^FOXP3^+^ T cell recovery following the high-dose chemotherapy (HDC) with autologous hematopoietic stem cell transplantation (auto-HSCT) in multiple myeloma (MM) patients.

Circulating CD4^+^FOXP3^+^ T cells of 79 MM patients were evaluated using flow cytometry before HDC with auto-HSCT, at the day of engraftment, and following 6 and 12 months.

Percentage of CD4^+^FOXP3^+^ T cells restored rapidly following auto-HSCT, became higher than pre-transplant level at the day of engraftment and then subsequently decreased for a year. CD4^+^FOXP3^+^ T cells at the time of engraftment were increased in patients with the relapse or progression of MM during 12 months following auto-HSCT (n=10) compared to non-relapsed patients (n=50): 6.7% (5.3—8.9%) vs 4.9% (2.8—6.6%); P_U_ = 0.026. Area under the curve was 0.72 (95% CI: 0.570—0.878; р=0.026). Circulating CD4^+^FOXP3^+^ T cell count was not associated with the percentage of myeloma plasma cells in a bone marrow but depended on its amount in autografts.

**Conclusions:**

Relative count of CD4^+^FOXP3^+^ T cells restored rapidly following auto-HSCT (at the day of engraftment), became higher than pre-transplant level and then subsequently decreased for a year. Their excess at the time of engraftment is associated with early relapse.

## INTRODUCTION

Multiple myeloma (MM) is a hematologic malignancy characterized by the uncontrolled proliferation of a B cell precursor clone that retains the ability to differentiate to plasma cells in the bone marrow (BM) and in most cases produces a pathological monoclonal immunoglobulin. The incidence of MM is about 1% of all cancers and it is the second most common hematologic malignancy after non-Hodgkin lymphoma [1, 2]. Due to the development and introduction of bortezomib, thalidomide, lenalidomide and high-dose chemotherapy (HDC) with autologous hematopoietic stem cell transplantation (auto-HSCT), the median overall survival of patients has increased to 5-7 years [3]. Nowadays active MM is one of the main indications for HDC with auto-HSCT, and is administered to all patients younger than 65 years in the absence of severe somatic pathology, but the relapse and disease progression are unavoidable [4].

The development of MM is associated with complex and still insufficiently studied interactions between tumor cells, BM stromal microenvironment and immunocompetent cells. Avoidance of malignant B cells from immune surveillance in hematological malignancies is mediated by a variety of mechanisms, among which an essential role is assigned to CD4^+^FOXP3^+^ regulatory T cells (Tregs), that are capable of suppressing the convenient T cell subsets [5]. However, research data on the changes in the count and functional activity of Tregs in MM remain controversial. Both a decrease [6] and an increase [7, 8] of Treg content in MM, as well as the lack of quantitative changes [9, 10] compared to healthy individuals were described. Some researchers found correlations between an increase of Treg counts and worsened survival rates [8, 11], in the other studies such associations were not identified [7, 9, 10].

The role of Tregs, as modulators of the antitumor immune response, appears to increase critically following auto-HSCT. HDC depletes the tumor clone in BM but simultaneously leads to prolonged lymphopenia and subsequent peripheral expansion of reinfused T cells. Unlike the main pool of convenient T cells, which had required about 6-12 months to recover, the percentage and absolute count of Tregs had reached relatively high values in the first month following transplantation [12–14].

Treg recovery following auto-HSCT was evaluated in MM; however, the data on the association of Treg count with the course of the disease after auto-HSCT were often opposite. It had shown both a gradual decrease of elevated Tregs [7, 8], and their increase to the values of healthy donors at initially lower relative amounts [13] in MM patients with remission following transplantation. The aim of the present work was to evaluate the dynamics of CD4^+^FOXP3^+^ T cell recovery in MM patients following HDC with auto-HSCT and the relationship between the CD4^+^FOXP3^+^ T cell counts and the course of the disease at the early post-transplant period.

## RESULTS

### Patient characteristics

Seventy-nine MM patients who had undergone the first HDC with auto-HSCT were enrolled in the retrospective study. Clinical characteristics of patients are described in Table 1. The median time from the diagnosis to auto-HSCT was 14.4 months (10.9—20.4 months). All patients treated with auto-HSCT had previously undergone conventional chemotherapy regimen. No thalidomide or lenalidomide were used before HDC. The majority of patients (n=70) were mobilized with either cyclophosphamide or other chemotherapeutic drugs plus granulocyte colony stimulating factor (G-CSF), seven patients received G-CSF alone and two individuals were mobilized following additional plerixafor administration. The median dose of CD34^+^ hematopoietic stem cells (HSCs) was 4.35 × 10^6^/kg (3.50—5.80 × 10^6^/kg). All patients received unmanipulated grafts. They underwent auto-HSCT after conditioning with melphalan 200 mg/m^2^ or 140 mg/m^2^. The median time of observation following auto-HSCT was 18.4 months (min-max: 1.1—63.7 months). Three-year overall survival and progression-free survival were 80% and 63%, respectively.

**Table 1:** Baseline characteristics of patients

Characteristic	Value
Age at auto-HSCT, years; median (min-max)	50 (32-64)
Sex, female/male	39/40
Types	
IgG	39
IgA	21
IgD	1
Light chain	11
Nonsecretory	1
NA	6
Durie-Salmon stage	
I	3
II	27
III	49
Disease status at auto-HSCT:	
- complete remission	23
- partial remission, very good partial response	43
- stable disease, progressive disease	13
Mobilization regimen:	
- Chemotherapy + G-CSF	70
• High-dose cyclophosphamide • CAV • DCEP	• 52 • 7 • 11
- G-CSF alone	7
- High-dose cyclophosphamide + G-CSF + plerixafor	2
Chemotherapy regimens before HDC with auto-HSCT	
1	40
2	24
≥3	15

Abbreviations: Auto-HSCT indicates autologous hematopoietic stem cell transplantation; CAV, cyclophosphamide, adriamycin, etoposide; DCEP, dexamethasone, cyclophosphamide, etoposide, cisplatin; G-CSF, granulocyte colony stimulating factor; HDC, high-dose chemotherapy; NA, not available.

### Dynamics of circulating CD4^+^FOXP3^+^ T cell recovery following auto-HSCT

**Figure 1:**
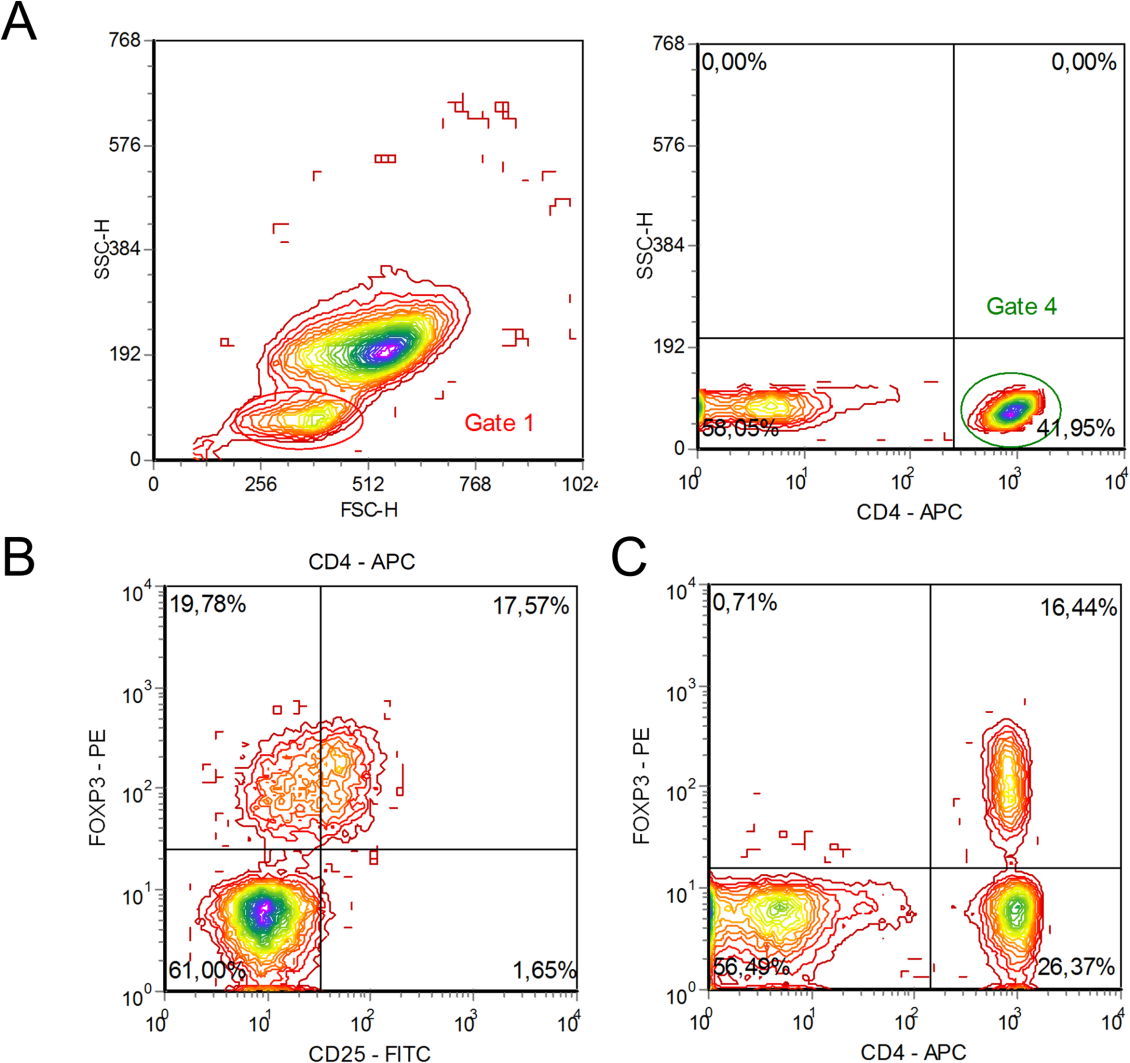
Flow cytometry characteristic of CD4^+^CD25^+^FOXP3^+^ regulatory T cells and CD4^+^FOXP3^+^ T cells in the peripheral blood of a multiple myeloma patient **(A)** Gating strategy of CD4^+^CD25^+^FOXP3^+^ and CD4^+^FOXP3^+^ T cell subsets is shown. **(B)** A relative count of FOXP3-positive CD4^+^ T cells among CD25-negative (upper left quadrant) and CD25-positive (upper right quadrant) populations is presented, percentage of CD4^+^ T cells. CD4^+^CD25^-^ (upper left quadrant) and CD4^+^CD25^+^ T cells (upper right quadrant) is not clearly divided into distinct regions. **(C)** A relative count of CD4^+^FOXP3^+^ T cells (upper right quadrant) in the lymphocyte gate. Flow cytometric analysis of a representative multiple myeloma patient is presented.

As mentioned above, there are still no definite data on a recovery of CD4^+^FOXP3^+^ T cells following HDC with auto-HSCT in MM patients. To study the post-transplant dynamics of Treg restoration, we evaluated relative and absolute counts of CD4^+^FOXP3^+^ T cells in the peripheral blood (PB) of MM patients before HDC, at the day of engraftment after auto-HSCT (on the average, + 14^th^ day, Figure 1) and following 6 and 12 months or until relapse or disease progression within this period.

Relative count of patient CD4^+^FOXP3^+^ T cells before HDC with auto-HSCT did not differ from that of healthy donors. During the first year after auto-HSCT, marked fluctuations were found. At the day of engraftment, CD4^+^FOXP3^+^ T cells were significantly higher compared with their count both in the healthy donors and in the patients before auto-HSCT. Subsequently, the percentage of CD4^+^FOXP3^+^ T cells decreased; six months after auto-HSCT it became significantly lower than the initial pre-transplant count and the donor values and remained at the same level till the end of the first post-transplant year (Table 2).

**Table 2:** Relative and absolute counts of lymphocytes, CD4^+^ T cells and CD4^+^FOXP3^+^ T cells in healthy donors and multiple myeloma patients before and following auto-HSCT

Cells	Healthy donors (n=27)	Before auto-HSCT (n=64)	Engraftment day (n=66)	6 months following auto-HSCT (n=35)	12 months following auto-HSCT (n=29)
Lymphocytes, × 10^9^/L	1.65 (1.47-2.09)	1.19^*^ (0.89-1.58)	0.69^*#^ (0.45-0.90)	1.52^#^ (1.07-2.18)	2.04^#^ (1.27-2.46)
CD4^+^ T cells,%	42.3 (37.7-49.4)	30.8^*^ (22.2-36.5)	22.8^*#^ (15.5-28.1)	18.8^*#^ (15.7-22.9)	22.3^*#^ (15.5-28.1)
CD4^+^FOXP3^+^ T cells,%	2.4 (2.0-3.3)	2.6 (1.7-4.2)	5.1^*#^ (3.1-7.0)	1.8^*#^ (1.2-2.3)	1.8^*#^ (1.0-2.4)
CD4^+^ T cells, /μL	637 (598-877)	293^*^ (214-407)	152^*#^ (101-274)	304^*^ (211-441)	299^*^ (275-503)
CD4^+^FOXP3^+^ T cells, /μL	47 (34-59)	29 (18-56)	30 (18-55)	27 (17-42)	27 (18-51)

Data are presented as median (interquartile range). *P* values are assessed with Mann–Whitney U-test.

^*^P_*U*_ < 0.05 between healthy donors and patients.

^#^*P*_*U*_ < 0.05 between patient values before and after auto-HSCT.

Auto-HSCT indicates autologous hematopoietic stem cell transplantation.

Simultaneously, there were no significant differences between absolute counts of CD4^+^FOXP3^+^ T cells before HDC and during the first year after auto-HSCT, as well as between the healthy donors` and the patients` values at all follow-ups (Table 2).

Relative counts of CD4^+^FOXP3^+^ T cells changed independently from CD4^+^ T cells during the post-transplant year. Percentages of CD4^+^FOXP3^+^ T cells and CD4^+^ T cells correlated with each other before HDC (r_S_=0.58, P=0.00036) and at the day of engraftment (r_S_=0.47, p=0.0019), while any correlations disappeared in 6 and 12 months following auto-HSCT (r_S_=0.20, p=0.41, and r_S_=0.41, p=0.10, respectively). Contrary to CD4^+^FOXP3^+^ T cell recovery, absolute count of CD4^+^ T cells remained decreased at the day of engraftment compared the pre-transplant patient level and did not reach the healthy control values during the observation period (Table 2).

### Association of elevated CD4^+^FOXP3^+^ T cell count at the day of engraftment with early post-transplant relapse or progression of MM

To evaluate possible association between CD4^+^FOXP3^+^ T cell recovery following auto-HSCT and the early relapse or progression of MM, we comparatively assessed the counts of these cells at the day of engraftment in patients in complete remission (CR) or in partial response (PR) and in relapsing individuals during the first post-transplant year. Among sixty patients who were observed more than one year after auto-HSCT, ten subjects had early disease relapse. The relapsing patients did not differ from the individuals in CR/PR by the age, the stage and the status of the disease, the type of immunoglobulin, the number of reinfused CD34^+^ HSCs (Table 3). A significant difference was found for the disease status at the time of HDC with auto-HSCT. The patients with stable disease or progressive disease had relapsed during the 1^st^ post-transplant year expectedly more often than the patients in CR or in PR/very good PR (Table 3).

**Table 3:** Characteristics of multiple myeloma patients depending on the course of the disease during the 1^st^ year following HDC with auto-HSCT

Characteristics	Complete or partial remission (*n* = 50)	Relapse or disease progression (*n* = 10)	P-value
Age at auto-HSCT, years; median (min-max)	50 (34–64)	52 (46–62)	0.45^a^
Sex, female/male	27/23	4/6	0.50^b^
Durie-Salmon stage			
I	2	1	0.43^b^
II	15	3	0.99^b^
III	33	6	0.73^b^
Types			
IgG	26	4	0.73^b^
IgA	18	3	0.99^b^
Light chain	6	3	0.16^b^
Disease status at auto-HSCT:			
- complete remission	16	1	0.26^b^
- partial response, very good partial response	31	5	0.50^b^
- stable disease, progressive disease	3	4	0.012^b^
Chemotherapy regimens before HDC with auto-HSCT			
1	31	5	0.50^b^
2	11	3	0.69^b^
≥3	8	2	0.67^b^
CD34^+^ hematopoietic stem cell dose, × 10^6^/kg	4,6 (3,6–6,0)	4,1 (3,5–4,5)	0.28^a^

Data are presented as median (interquartile range) or number of patients. *P* values are assessed with ^a^Mann–Whitney U-test and ^b^Fisher exact test.

Auto-HSCT indicates autologous hematopoietic stem cell transplantation; HDC, high-dose chemotherapy.

**Figure 2:**
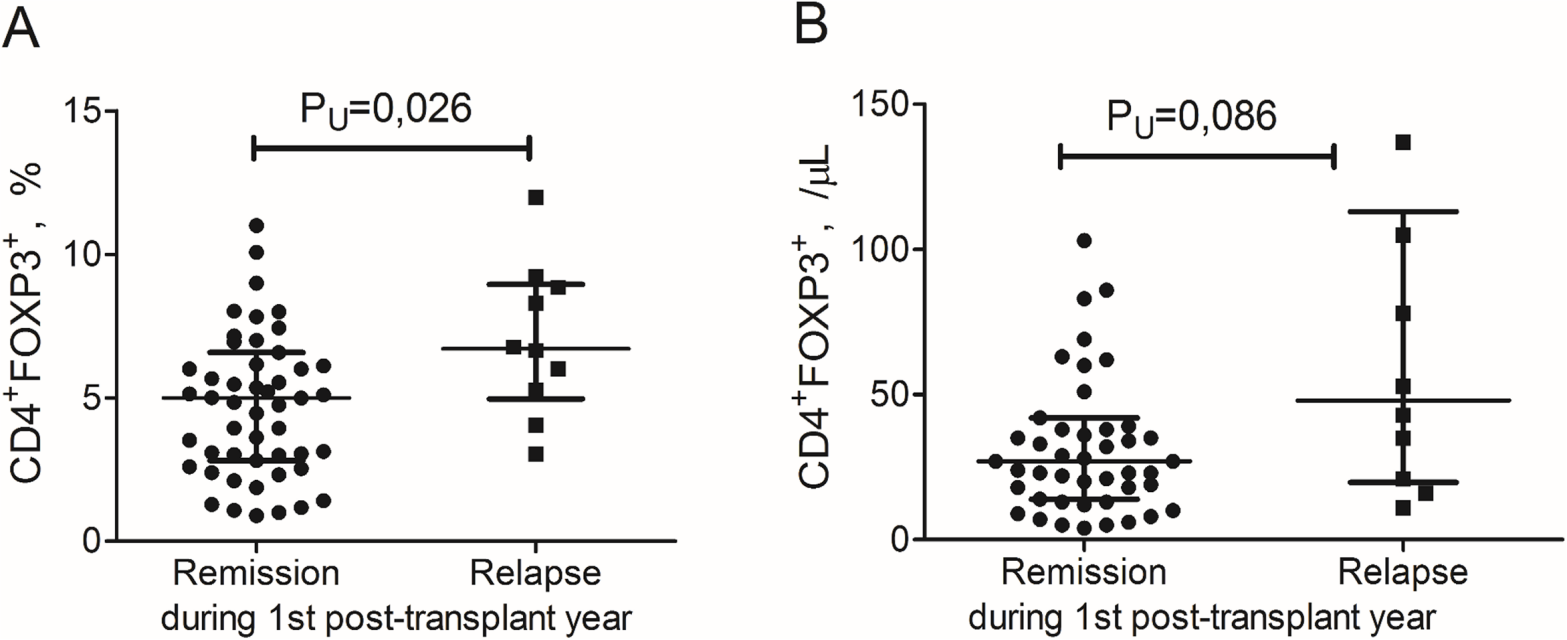
CD4^+^FOXP3^+^ T cells in the peripheral blood of multiple myeloma patients at the day of engraftment depending on the course of the disease during the 1^st^ post-transplant year Individual values of relative **(A)** and absolute **(B)** counts of CD4^+^FOXP3^+^ T cells are presented. Lines and scatter plots show the medians and interquartile ranges. *P* values are assessed with Mann–Whitney U-test.

Higher relative count of CD4^+^FOXP3^+^ T cells at the day of engraftment was observed in the patients with early relapse or progression of MM compared to non-relapsing patients: 6.7% (5.3—8.9%) vs 4.9% (2.8—6.6%); P_U_ = 0.025 (Figure 2A). There was a non-significant trend between these groups in the absolute CD4^+^FOXP3^+^ T cell count: 48 /μL (21—105 /μL) vs 27 /μL (14—39 /μL); p_U_ = 0.088 (Figure 2B). There were no any significant differences between the relapsing and non-relapsing patients in absolute lymphocyte count (0.72 × 10^9^/L (0.39—1.13 × 10^9^/L) vs 0.67 × 10^9^/L (0.49—0.90 × 10^9^/L); p_U_=0.75) and relative and absolute CD4^+^ T cell counts at the day of engraftment (31.7% (19.1—34.3%) vs 22.8% (17.4—32.1%); p_U_=0.67, and 254 /μL (94—432 /μL) vs 276 /μL (134—420 /μL); p_U_=0.74, respectively).

### Predictive value of circulating CD4^+^FOXP3^+^ T cells for early relapse in MM

**Figure 3:**
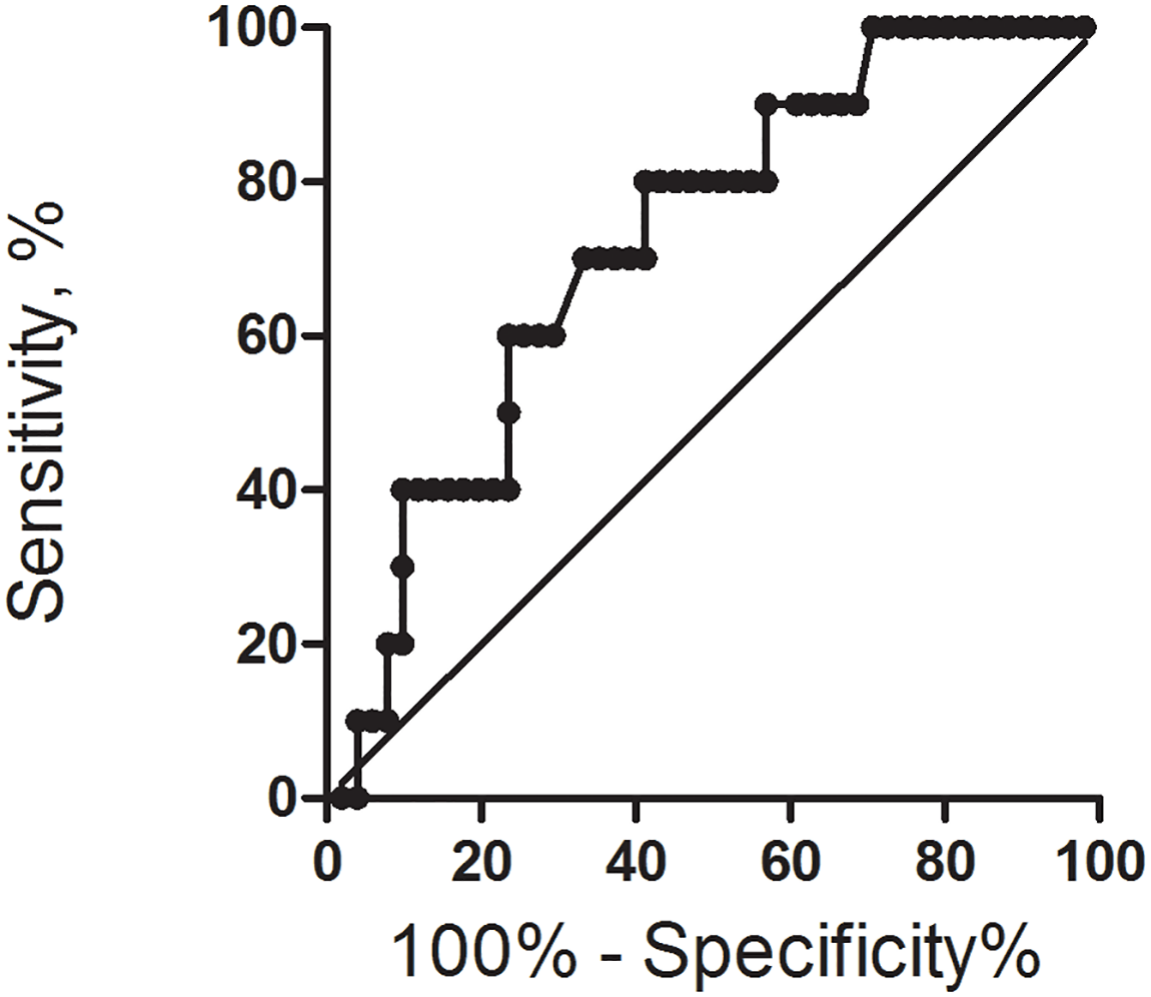
Receiver operating-characteristic curve and area under the curve (AUC) for relative CD4^+^FOXP3^+^ T cell count at the day of engraftment in 1^st^ year progression-free survival analysis

To determine a significance of the relative count of CD4^+^FOXP3^+^ T cells as a predictive factor for MM course during the 1^st^ year following auto-HSCT, ROC analysis was performed. ROC curve and associated AUC analysis showed that the percentage of CD4^+^FOXP3^+^ T cells at the day of engraftment was a significant marker for early relapse or progression in MM patients: AUC was 0.72 (95% CI: 0.570—0.878; р=0.026) (Figure 3). CD4^+^FOXP3^+^ T cell relative count > 5.84% allowed to predict early relapse (70.0% sensitivity, 68.0% specificity, with 2.19 likelihood ratio).

Using the obtained cut-off of 5.84% for CD4^+^FOXP3^+^ T cells at the day of engraftment, the baseline characteristics of patients were compared. There were no differences between patients having high (> 5.84%) vs low (≤ 5.84%) relative count of CD4^+^FOXP3^+^ T cells by the age, the stage and the status of the disease, the type of immunoglobulin and the number of reinfused CD34^+^ HSCs (Table 4).

**Table 4:** Baseline characteristics of multiple myeloma patients depending on the calculated cut-off point for circulating CD4^+^FOXP3^+^ T cells at the day of engraftment

Characteristics	≤ 5.84% (*n* = 37)	>5.84% (*n* = 23)	P-value
Age at auto-HSCT, years; median (min-max)	50 (34–62)	53 (36–64)	0.12^a^
Sex, female/male	22/15	9/14	0.18^b^
Durie-Salmon stage			
I	2	1	0.99^b^
II	13	6	0.57^b^
III	22	16	0.58^b^
Types			
IgG	16	13	0.43^b^
IgA	16	6	0.27^b^
Light chain	5	4	0.72^b^
Disease status at auto-HSCT:			
- complete remission	11	5	0.56^b^
- partial response, very good partial response	22	14	0.99^b^
- stable disease, progressive disease	4	4	0.47^b^
Chemotherapy regimens before HDC with auto-HSCT			
1	21	15	0.59^b^
2	9	5	0.99^b^
≥3	7	3	0.73^b^
CD34^+^ hematopoietic stem cell dose, × 10^6^/kg	4,6 (3,6–6,8)	4,3 (3,3–5,7)	0.46^a^

Data are presented as median (interquartile range) or number of patients. *P* values are assessed with ^a^Mann–Whitney U-test and ^b^Fisher exact test.

Auto-HSCT indicates autologous hematopoietic stem cell transplantation; HDC, high-dose chemotherapy.

### Lack of direct relationship between BM myeloma plasma cells or the course of the disease at the time of HDC with auto-HSCT and circulating CD4^+^FOXP3^+^ T cell count

Further, we tried to assess a possible direct impact of BM myeloma plasma cells (PCs) on the counts of peripheral blood (PB) CD4^+^FOXP3^+^ T cells. Relative count of BM CD45^dim^CD38^+^CD138^+^CD56^+^CD19^—^CD117^+^CD27^—^CD81^—^ malignant PCs in 32 MM patients before HDC or in 6 and 12 months following auto-HCST was evaluated simultaneously with CD4^+^FOXP3^+^ T cells. As anticipated, myeloma PCs were significantly higher in patients with the relapse or progression of the disease compared to individuals in CR or PR (Table 4). At the same time, there were no differences in relative and absolute counts of PB CD4^+^FOXP3^+^ T cells between the patients without detectable levels (<0.01% of all nucleated BM cells) of myeloma PCs and the patients with minimal residual disease (Table 5). Also, relative and absolute counts of PB CD4^+^FOXP3^+^ T cells did not correlate with the relative count of BM myeloma PCs: r_S_=0.17, p=0.35, and r_S_=0.11, p=0.55, respectively.

**Table 5:** Counts of BM myeloma plasma cells and PB CD4^+^FOXP3^+^ T cells according to the course of the disease

Cells	Complete remission MRD-negative (n=9)	Complete remission MRD-positive (n=10)	Partial response (n=8)	Stable disease, progressive disease, relapse (n=5)
Myeloma PCs,% of all nucleated cells	–	0.147^*^ (0.060-0.371)	0.103^*^ (0.074-0.216)	2.099 (1.635-3.000)
CD4^+^FOXP3^+^ T cells,%	1.9 (1.4-2.0)	1.2 (1.1-1.8)	2.4 (1.1-4.4)	2.0 (1.6-2.1)
CD4^+^FOXP3^+^ T cells, /μL	21 (19-27)	18 (14-28)	24 (14-32)	37 (29-44)

Data are presented as median (interquartile range). *P* values are assessed with Mann–Whitney U-test.

^*^P_*U*_ < 0.005 compared to patients in stable disease, progressive disease and relapse.

MRD was defined if myeloma PCs were ≥ 0.01% of all nucleated cells.

BM indicates bone marrow; MRD, minimal residual disease; PB, peripheral blood; PCs, plasma cells.

Similarly, no significant differences were found between MM patients in CR, PR and stable or progressive disease when assessing relative or absolute CD4^+^FOXP3^+^ T cell counts in PB before HDC and at the day of engraftment following auto-HSCT (Table 6).

**Table 6:** Counts of PB CD4^+^FOXP3^+^ T cells according to the course of the disease

Cells	Complete remission (n=13 and n=16)	Partial response (n=32 and n=38)	Stable disease, progressive disease (n=8 and n=9)	P-value
CD4^+^FOXP3^+^ T cells before HDC,%	2.0 (1.2-3.2)	3.2 (1.8-4.6)	2.1 (1.9-3.7)	0.43
CD4^+^FOXP3^+^ T cells at the day of engraftment,%	4.6 (3.1-6.5)	5.3 (3.0-7.0)	5.3 (4.1-8.0)	0.95
CD4^+^FOXP3^+^ T cells before HDC, /μL	18 (15-47)	32 (19-62)	28 (24-55)	0.42
CD4^+^FOXP3^+^ T cells at the day of engraftment, /μL	28 (22-36)	28 (13-53)	43 (19-78)	0.61

Data are presented as median (interquartile range). *P* values are assessed with Kruskal–Wallis test.

HDC indicates high dose chemotherapy; PB, peripheral blood.

### Impact of reinfused autograft CD4^+^FOXP3^+^ T cells on their count at the day of engraftment

Previously it was shown, that early recovery of lymphocytes after HDC with auto-HSCT is dependent on the peripheral expansion of reinfused cells in MM patients [15, 16]. In our study, absolute CD4^+^FOXP3^+^ T cells count at the day of engraftment was higher in patients who underwent two or more leukapheresis procedures compared to those with a single HSC harvest: 36 /μL (25—60 /μL, N=25) vs 27 /μL (11—51 /μL, N=38); P_U_=0.044.

To study the association between the dose of reinfused CD4^+^FOXP3^+^ T cells and their count at the day of engraftment, we assessed absolute count of these cells in autografts of patients who underwent a single leukapheresis procedure and therefore were further transplanted with only one cryobag. Under above conditions leukapheresis samples of twenty patients were available. The median dose of reinfused CD4^+^FOXP3^+^ T cells was 3.52 × 10^6^/kg (2.09—4.90 × 10^6^/kg), and their absolute count at the engraftment day was 23 /μL (10—54 /μL). Autograft and PB absolute CD4^+^FOXP3^+^ T cell counts correlated with each other (r_S_=0.61, P=0.00012). A logistic regression analysis using the cutoff value of the median (30 /μL) for PB CD4^+^FOXP3^+^ T cell counts at the day of engraftment indicated that higher absolute count in autograft predicted their more effective recovery as well (odds ratio (OR): 13.50, P = 0.0014). The logistic regression models for predicting CD4^+^FOXP3^+^ T cell recovery did not reveal any prognostic values for autograft lymphocytosis and CD34^+^ HSCs (OR: 1.57, p=0.19 and OR: no, p=0.50, respectively), however, higher absolute count of autograft CD4^+^ T cells was associated with more effective CD4^+^FOXP3^+^ T cell recovery as a non-significant trend (OR: 3.56, p = 0.051).

## DISCUSSION

MM is an incurable malignant disorder considered to be the result of an uncontrolled proliferation of a B cell precursor clone in the bone marrow and more rarely in soft tissues; malignant cells further differentiate to mature PCs. Search for reproducible predictive methods continues, and the main goal is still creating an opportunity for successful therapeutic intervention. Given the pathogenesis of the disease, the most likely prognostic markers may be immune response parameters that reflect the nature of immune recovery and the quality of the antitumor response.

Malignant cell avoidance of immune surveillance in MM is promoted by activation of different populations of immune and stromal cells with regulatory activity e.g. Tregs, myeloid-derived suppressor cells, mesenchymal stromal cells etc. In turn, the term “regulatory T cells” includes different subsets of T lymphocytes capable of suppressing the immune response. Nowadays the most well-known population is CD4^+^ Tregs co-expressing CD25 molecule and FOXP3 transcription factor. However, FOXP3 and other surface and intracellular markers, as well as their absence, are not specific for Tregs, since these molecules are expressed by activated T cells. On the contrary, several subsets of CD4^+^ Tregs are CD25-negative, but possess certain – more or less – suppressive potential [17–20]. Therefore, in the present work we retrospectively studied the recovery dynamics of CD4^+^ T cell subset with intracellular expression of FOXP3 in MM patients, who underwent HDC with auto-HSCT. The studying subset may include some quantity of activated convenient T cells that actually not suppressive, but hyporesponsive [21]. To avoid misunderstanding, we did not define the studying subset as “Tregs” in the text.

The obtained results indicate that the relative count of CD4^+^FOXP3^+^ T cells in PB of MM patients varies notably during the first year after HDC with auto-HSCT. Unlike CD4^+^ T cells, CD4^+^FOXP3^+^ T cells significantly exceeded both pre-transplant and healthy donor values at the day of engraftment, and thereafter decreased during the year until the patient values became lower than healthy donor ones had been. The rapid recovery of PB CD4^+^FOXP3^+^ T cells after HSCT is consistent with previously obtained data [12–14]. Earlier several researchers showed an increase in Treg proliferative activity after allogeneic HSCT [12, 14, 22]. At the same time, the absolute count of PB CD4^+^FOXP3^+^ T cells remains at the pre-transplant level from the day of engraftment to the end of the first year after auto-HSCT, due to gradual increase of absolute lymphocyte count.

The role of a rapid recovery of Tregs on the conditions of lymphopenia and their influence on peripheral lymphocyte expansion remain subject of debate. S. Shen et al. [23] demonstrated that murine Tregs more effectively suppressed homeostatic proliferation of T cells with low affinity of T cell receptors. C. Winstead et al. [24] showed that CD4^+^CD25^+^FOXP3^+^ T cells inhibited an antigen-specific “spontaneous” expansion of T lymphocytes, but did not affect the homeostatic proliferation induced by interleukin 7. The same authors later noted that the absence of Tregs during the period of intensive T cell recovery led to the limitation of T cell receptor repertoire and to the decreased immune response to foreign antigens [25]. It`s possible that Tregs prevent an uncontrolled expansion of rapidly proliferating clones, thereby contributing to a future variety of T cells.

About 20% of MM patients relapse within 12 months following auto-HSCT, and this is a significant predictor of poor overall survival [26]. We aimed to evaluate an association of early relapse of the disease with CD4^+^FOXP3^+^ T cells during early post-transplant period (<12 month following auto-HSCT). Our study demonstrated that a higher relative CD4^+^FOXP3^+^ T cell count in the early post-transplant period was associated with MM relapse or progression within 12 months after an auto-HSCT. The earlier obtained data on the relationship between Tregs and outcomes of auto-HSCT is ambiguous. Thus, according to the data of K.R. Muthu Raja et al. [8] and K. Giannopoulos et al. [11], the increased amount of CD4^+^CD25^+^FOXP3^+^ Tregs in MM is associated with poor prognosis. At the same time, there was no correlation between the count of Tregs and survival rates in several other studies [7, 9, 10]. However, the enumerated studies included relatively low numbers of patients who underwent auto-HSCT (5--26 individuals) and their data generally were assessed without distinguishing from non-transplanted groups.

It is difficult to say whether the rise of PB CD4^+^FOXP3^+^ T cells in early post-transplant period was the cause or consequence of the progression of MM. We did not find any associations between the relative count of residual myeloma PCs in BM and the amount of PB CD4^+^FOXP3^+^ T cells. Malignant plasma cells, derived from patients and MM cell lines, are able to stimulate the generation of inducible Tregs [27, 28], but under the conditions of substantial tumor cell depletion after both conventional chemotherapy regimens and HDC the possibility of a tumor-inducing Treg generation is limited. Presumably, a higher relative count of CD4^+^FOXP3^+^ T cells itself, under the lymphopenic conditions, can lead to a restriction of effector T cell expansion. The subsequent lack and/or decreased function of T cells can promote the progression of MM.

According to previous studies focused on prevention of graft-versus-host-disease following allogeneic HSCT in hematologic malignancies, it is known that the post-transplant recovery of Tregs depends on their count in the graft [14, 29, 30]. Here we confirmed that the main source of CD4^+^FOXP3^+^ T cells under severe lymphopenic conditions immediately after auto-HSCT is the same T cell subset reinfused together with CD34^+^ HSCs. The higher amount of graft CD4^+^FOXP3^+^ T cells was associated with the increased absolute count of this T cell subset. However, we did not find any difference in the studying group between the relapsing (N=5) and non-relapsing MM patients (N=15) during the 1^st^ year following auto-HSCT in absolute autograft CD4^+^FOXP3^+^ T cell counts, possibly due to a small group size (data not shown).

The performed ROC analysis showed that the increased relative count of CD4^+^FOXP3^+^ T cells at the day of engraftment was a significant marker for early relapse or progression in MM patients. The detected association of circulating CD4^+^FOXP3^+^ T cells and survival rates is modest (AUC was 0.72) and hardly can be used as an exclusive predictor of early relapse after auto-HSCT. Nevertheless, a flow cytometric evaluation of this subset is relatively simple and reproducible; usually there is no difficulty in dividing the cell region of interest (Figure 1C). Cytogenetic and FISH methods have a good prognostic significance [31], but still are inaccessible in a lot of regions.

Thus, higher count of CD4^+^FOXP3^+^ T cells in PB following auto-HSCT is associated with unfavorable MM prognosis. Further studies will provide a better understanding of the interaction between the tumor, immune cells and stromal microenvironment and may shed a light on the role of Tregs in determining the course of the disease in MM.

## MATERIALS AND METHODS

### Patients and healthy donors

Seventy-nine MM patients who had undergone HDC with auto-HSCT between October 2009 and October 2017 at Research Institute of Fundamental and Clinical Immunology (Novosibirsk, Russia) and 27 age-matched and gender-matched healthy donors were enrolled in the retrospective study. All healthy subjects and patients gave informed consent in accordance with the Declaration of Helsinki of 1975. The local ethics committee approved the study protocol. The inclusion criteria were: indications for HDC with auto-HSCT; the first auto-HSCT; a sufficient count of collected CD34^+^ HSCs; an adequate organ function (except of myeloma-induced renal failure). Exclusion criteria were: any major organ failure, severe drug hypersensitivity, active infectious diseases, major autoimmune diseases. Patients were staged according to the Durie-Salmon system (1975). Responses were defined according to International Myeloma Working Group criteria. Early relapse was defined as relapse within 12 months post-transplant. We evaluated relative and absolute counts of several T cell subsets in the peripheral blood (PB) of MM patients before HDC (n=53), at the day of engraftment after auto-HSCT (n=63) and following 6 (n=28) and 12 months (n=22). The number of patients changed at the follow-ups because of inability to obtain blood samples; additionally, the patients received chemotherapy because of relapse or second auto-HSCT were subsequently excluded from the study but their data from previous follow-ups remained.

### Blood and graft samples

Venous heparinized blood samples obtained from healthy donors were a part of blood donations. Blood samples from patients were obtained during routine diagnostic procedures. Graft samples were obtained during the cryopreservation procedure. Data of patient peripheral blood cell counts and apheresis product cell counts were obtained from medical records. Mononuclear cells (MNCs) were isolated by density gradient centrifugation using Ficoll-Paque (Pharmacia Biotech, Uppsala, Sweden), washed with phosphate buffer solution and with RPMI-1640 Medium supplemented with 0.3 g/L L-glutamine, 25 mM HEPES (Sigma-Aldrich, St. Louis, MO, USA), 100 μg/mL gentamicin (Sigma-Aldrich), 10% autologous serum and underwent immediate analysis by flow cytometry. Graft samples were frozen at –80°C and subsequently thawed immediately before analysis.

### Flow cytometric analysis of T cell subsets

Freshly isolated or thawed MNCs were stained with the following mouse anti-human monoclonal antibodies according to the manufacturer`s recommendations: CD4-fluorescein isothiocyanate (FITC, clone L200, BD Biosciences, San Jose, CA, USA) or peridinin chlorophyll protein (PerCP, clone L200, BD Biosciences), or allophycocyanin (APC, clone L200, BD Biosciences); FOXP3-phycoerythrin (РЕ, clone 259D/C7, BD Biosciences). For intracellular staining, samples were incubated with commercially available Transcription Factor Buffer Set (BD Biosciences) according to manufacturer instructions. The studied T cell subset were defined as the CD4^+^FOXP3^+^ population regardless of the expression CD25 molecule, since FOXP3 transcription factor had been detected both in CD4^+^CD25^hi^ subtype and in CD4^+^CD25^-^ T cells (Figure 1). During the time of the study, samples were analyzed on two FACSCalibur flow cytometers and FACSCanto II flow cytometer (BD Biosciences) using CellQuest Pro and FACSDiva software, respectively (BD Biosciences). Lymphocytes were first gated based on forward and side scatter. At least 30,000 gated CD4^+^ T cells per sample were collected for the assay. Both CD4^+^ T cells and CD4^+^FOXP3^+^ T cells were presented as the percentages of lymphocytes.

### Flow cytometric assessment of myeloma plasma cells

Fresh BM samples were stained with the following mouse anti-human monoclonal antibodies according to the manufacturer`s recommendations: CD38-FITC or APC, CD56-PE, CD27-peridinin chlorophyll protein complex with cyanin-5.5 (PerCP-Cy5.5), CD138-PerCP-Cy5.5 or APC, CD117- phycoerythrin-cyanin-7 (Pe-Cy7), CD81-allophycocyanin-Hilite® 7 (APC-H7), CD19-V450, CD45-V450, cytIgLambda-FITC, cytIgKappa-PE-Cy7 (all MAbs were obtained from BD Biosciences). For intracellular staining, samples were incubated with commercially available BD FACS Permeabilizing Solution 2 (BD Biosciences) according to manufacturer`s instructions. Samples were analyzed on FACSCanto II flow cytometer (Becton Dickinson) using FACSDiva software, respectively (Becton Dickinson). The instrument was daily calibrated with calibration beads (Cytometer Setup and Tracking from BD Biosciences). At least 10^6^ gated BM cells per sample were collected for the assay to attain the sensitivity 0.01% (i.e. 10^-4^). Myeloma PCs were indicated as CD45^dim^CD38^+^CD138^+^CD56^+^CD19^—^CD117^+^CD27^—^CD81^—^ phenotype gate and were presented as the percentage of all nucleated BM cells. Gating strategy is presented in Supplementary Figure 1.

### Statistical analysis

Statistical analysis was performed using Statistica 6 (StatSoft) package. Data were presented as median with minimum-maximum or interquartile ranges. Fisher exact test were used to determine relationships between categorical variables. The Mann-Whitney U test and Kruskal–Wallis test were used to calculate differences between groups of patients. Spearman’s rank correlation was used to evaluate associations for continuous variables. Logistic regression was used to measure the influence of the independent variable (absolute autograft CD4^+^FOXP3^+^ T cell count) upon the dependent variable (lower or higher absolute PB CD4^+^FOXP3^+^ T cell count at the day of engraftment). To determine a predictive significance ROC analysis was performed. P values presented were two-sided. P < 0.05 was considered statistically significant. Statistical analysis was performed using Statistica 6 (StatSoft, USA) package. Graphs were prepared using GraphPad Prism 5 software (GraphPad Software, Inc., USA).

## SUPPLEMENTARY MATERIALS FIGURE



## Abbreviations

Auto-HSCT: indicates autologous hematopoietic stem cell transplantation
BM: bone marrow
CAV: cyclophosphamide, adriamycin, etoposide
CR: complete remission
DCEP: dexamethasone, cyclophosphamide, etoposide, cisplatin
G-CSF: granulocyte colony stimulating factor
HDC: high-dose chemotherapy
MM: multiple myeloma
MRD: minimal residual disease
OR: odds ratio
PB: peripheral blood
PC: plasma cell
PR: partial response
P_U_: statistical significance calculated with The Mann-Whitney U test
ROC: receiver operating characteristic
TCR: T cell receptor
Treg: regulatory T cell
