# Supplementary material for: Increased circulating CD4+FOXP3+ T cells associate with early relapse following autologous hematopoietic stem cell transplantation in multiple myeloma patients
Source: Oncotarget. 2018 Jun 5;9(43):27305–17. doi: 10.18632/oncotarget.25553 (PMC6007464; doi:10.18632/oncotarget.25553)
Supplement: Supplementary file 1 [file oncotarget-09-27305-s001.pdf]

Increased circulating CD4⁺FOXP3⁺ T cells associate with early relapse following autologous hematopoietic stem cell transplantation in multiple myeloma patients

SUPPLEMENTARY MATERIALS

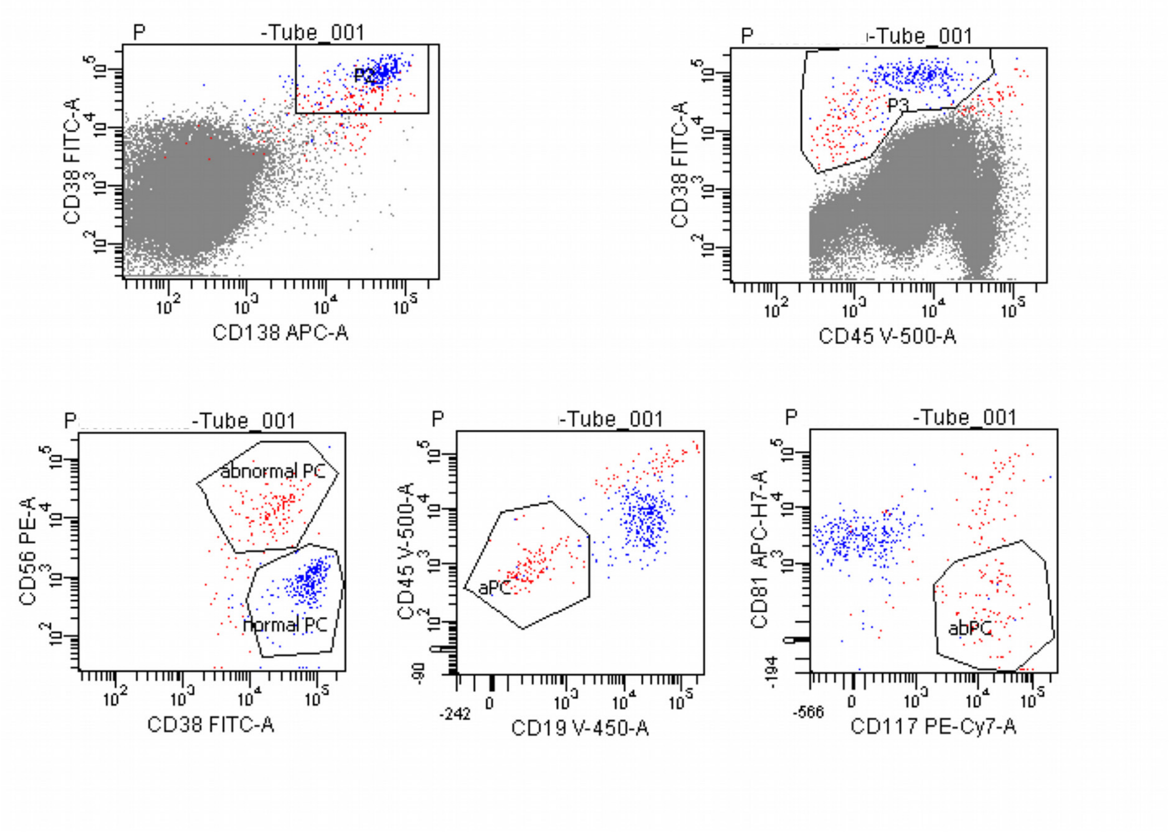

	FITC	PE	PerCP-Cy5.5	PE-Cy7	APC	APC-H7	V450	V510
Tube 1	*CD38*	*CD56*	*CD27*	*CD117*	*CD138*	*CD81*	*CD19*	*CD45*
Tube 2	*cyt IgL*	*CD56*	*CD138*	*cyt IgK*	*CD38*		*CD19*	*CD45*

Supplementary Figure 1: Gating strategy and 8-colour monoclonal antibody cocktail for distinguishing normal and myeloma bone marrow plasma cells. Flow cytometric analysis of a representative patient is presented.
